# Supplementary material for: Are carbon emissions trading and green financial instruments synergistic? -Comprehensive quantitative research based on content analysis
Source: PLoS One. 2024 Mar 7;19(3):e0298601. doi: 10.1371/journal.pone.0298601 (PMC10919601; doi:10.1371/journal.pone.0298601)
Supplement: S1 Data — (ZIP) [file pone.0298601.s001.zip › Data/地级市Z_F工作报告.zip!/║╙▒▒/▓╫╓▌╩╨/└┤╫╘╣1⁄2╓┌║┼í╛┬φ┐╦╩2╛▌═°í┐_2005.docx]

各位代表：

现在，我代表市人民政府，向大会作工作报告，请予审议，并请各位政协委员和其他列席会议的人员提出意见。

一、2004年工作回顾

过去的一年，是我市改革发展进程中不平凡的一年，也是经济建设和社会发展取得显著成就的一年。在省委、省政府和市委的正确领导下，我们全面贯彻党的十六大和十六届三中、四中全会精神，以科学发展观为指导，统筹经济和社会发展，团结带领全市人民迎难而上，顽强拼搏，圆满完成了市十一届人大二次会议确定的各项工作任务，为沧州的大发展、快发展奠定了坚实的基础。

（一）经济发展呈现出迅猛增长的好势头

——发展速度明显加快，主要经济指标全面攀升。初步核算，2004年全市生产总值完成774.1亿元，比上年增长15.3％，生产总值的年增长首次突破100亿元，提前一年超额完成“十五”计划目标。其中，第一产业增加值111.3亿元，第二产业增加值400.9亿元，第三产业增加值261.9亿元，分别增长17％、16.4％和13％。全社会固定资产投资完成243.7亿元，增长40.5％，首次突破200亿元大关。全部财政收入完成65.1亿元， 增长21.8％，其中一般预算收入完成25.8亿元，增长19.2％。

——经济运行质量提高，工业在调整中快速增长。新增规模以上企业24家，规模以上工业企业完成增加值130亿元，销售收入497亿元，实现利润22.4亿元，分别增长29.1％、31.8％和59.3％，拉动全市经济增长5.2个百分点。国家统计局公布的全国1948家大型工业企业名单中，我市沧炼、沧化、大化、供电公司、东塑5家企业榜上有名。全市入统工业产品产销率达到98.9％，高出上年1.16个百分点，出现产销两旺的好形势。全市规模以上工业累计完成新产品产值4.85亿元，是上年的1.95倍，一批科技含量高、成长性好的新产品开发和技术改造项目走向成熟。

——投资、出口拉动增长强劲，发展后劲持续增强。在国家加强宏观调控的新形势下，我们坚持在调整中适应，在适应中发展，进一步优化投资结构，投资和项目建设保持了强劲增势。全社会固定资产投资打破了多年来低于全省平均水平的局面。规模以上工业新增固定资产16亿元，是上年新增固定资产的1.96倍。全年完成出口创汇4.83亿美元，增长62.2％，出口净增1.85亿美元，拉动经济增长2.28个百分点。

（二）重点工作取得新突破，带动和活跃了经济发展全局

——对外开放迈出坚实步伐。对外开放氛围日益浓厚，招商引资工作机制更加完善，利用外资领域逐步拓宽。专业招商和小团组“一对一”招商效果明显。新签“三资”项目合同82个，增长51.9％，合同总额30182万美元，增长25.6％，合同利用外资17512万美元，增长76％。直接利用外资8276万美元，比上年增长173.4％，创历史最高。沧化集团与香港华士达化工集团达成合资建设12万吨己内酰胺项目的意向，标志着全市对外开放攀上新的台阶。同时，加强了与京津及周边地区的经济技术合作，引进省外资金50.7亿元，引进技术712项。

——项目建设取得重要突破。落实中央宏观调控政策，对土地、投资等领域存在的问题进行了认真清理。按照国家产业政策，紧紧围绕主导产业、优势产业、特色产业搞好项目谋划和建设，项目建设呈现出良性发展的好局面。全市投资1000万元以上的重点项目1372个，总投资1575.1亿元，比上年增加426个和319.6亿元。在建项目506个，其中新开工项目329个，分别增加200个和82个。竣工项目348个，比上年增加81个。其中亿元级以上大项目达到265个，总投资1293.6亿元，一批对全市经济有重要影响的大项目相继开工建设。黄骅港煤码头二期工程投入试运行，朔黄铁路复线开通；环保微生物制剂、远东高密度互联多层板等项目竣工投产；国华沧东电厂、TDI“2改3”、40万吨PVC、液体化学品码头及罐区、惠丰塑料电缆料等项目进展顺利。

——民营经济在发展中壮大。进一步放宽政策，降低民营企业进入门槛，削减企业注册登记审批项目近1/3。加强信贷担保中心建设，形成融资担保能力4亿元，有力地支持了民营经济发展。通过扶优扶强，进一步壮大电线电缆、弯头管件、线路板、裘革制品、塑料制品、农副产品加工等重点优势产业，形成一批在国内占据重要位置的特色产业集群。全市民营经济完成增加值494.2亿元，增长25.4％，民营经济增加值占国内生产总值的比重达到63.9％，成为支撑全市经济增长的重要力量。

——各项改革稳步推进。牢牢抓住产权制度改革这个核心，加大国有企业改革推进力度，全市1115家国有企业已完成改制743家，改制面为67％。其中，实施破产235家，兼并72家。重点企业产权制度改革有了新的突破。沧运集团改制顺利完成，地铁“双置换”稳步推进。市直42家工业危困企业已有31家进入实质性运作阶段，核销呆坏帐14.3亿元，盘活闲置资产3.94亿元，解除担保10.2亿元。初步建立了国资委、国有资产经营公司、企业三个层次的监督管理体系，国有资产监管得到加强。粮食系统改革稳步推进，财税改革、事业单位改革取得初步进展。

——“三农”工作取得重要进展。农业基础地位进一步巩固，农村经济全面发展。认真贯彻落实中央一号文件和各项惠农政策，对种粮农民直接补贴资金7779万元，调动了广大农民发展粮食生产的积极性，全市粮食总产量达323.8万吨，增长23.7％。生态林建设和果品生产进一步加强，完成造林47万亩，果品总产量达10.5亿公斤。农业结构调整取得新成效，“四大产业、一大工程”产值已占全市农业总产值的56％以上，成为农村经济的重要支撑和农民增收的新渠道。产业化龙头企业不断壮大，又有青县小洋人、河北乡谣等7家企业进入省级农业产业化龙头企业行列，使我市省级龙头企业总数达到16家。积极做好“禽流感”防治工作，畜牧业保持平稳增长。全市肉蛋奶总产分别达到42.1万吨、31.5万吨和7.4万吨，增长10.9％、4％和41.2％。

（三）城乡面貌发生新变化，人民生活水平进一步提高

——民心项目如期完成。坚持把提高和改善群众生产生活条件作为执政为民的首要内容，从解决群众最现实、最关心、最直接的问题入手，精心组织实施了十二个“民心项目”。“引大入港”工程完成投资1.2亿元，铺设输水管道80.3公里，于去年12月正式向临港化工园区供水；市区新增供热面积40万平方米，发展天然气用户7900户；投资1800万元，新建、改造市区公厕259座；投资3.35亿元，新铺油路1656公里，新打和维护深浅机井19眼，解决了18个少数民族村交通和饮水问题；创建信用乡镇24个、信用村2025个，信用户36.5万户，新增小额信贷10.4亿元，促进了贫困村、贫困户稳定脱贫。

——城乡基础设施建设加快。以改善生产条件和人居环境为目标，重点实施了一批交通、排水和污水处理项目。完成了新华路、新华桥、北环桥等路桥改造工程，对市区解放路、西环路等重要路段实行雨污分流，新修排水管线5公里，新建改建泵站7座，新增排水能力7立方米/秒，提高了市区防洪能力。10万吨污水处理厂、400吨/日垃圾处理厂项目均已完成并投入使用。市区污水处理率达到70％，生活垃圾无害化处理率100％。沧黄、津汕、保沧高速公路已开工建设，阿深及沧东绕城高速公路前期工作进展顺利。新建、改建县乡道路187公里。“村村通”工程完成投资1.66亿元，相当于我市前十年村级公路建设投资总和，为980个村修通了油路，总里程1471公里，极大地改善了农村交通条件。开展水污染专项整治，加强湿地保护。海兴湿地和鸟类自然保护区已建成省级自然保护区；南大港湿地正在申报国家级自然保护区。

——劳动就业和社会保障工作进一步加强。大力开辟就业渠道，全市新增就业岗位51500个，下岗失业人员再就业18700人。全市城镇登记失业率3.48％，低于省3.5％的指标。向京津等大中城市输出劳务33.8万人，向境外输出劳务780人。认真清理解决拖欠农民工工资问题，清理发还农民工工资4335万元。建立了建设单位对农民工直接发放工资制度，有效避免形成新的拖欠。社会保障覆盖面不断扩大，企业养老保险和职工基本医疗保险制度改革积极推进。为企业离退休人员发放养老金52154.3万元，按时发放和社会化发放率均达到100％。

——城乡居民收入大幅增加，生活条件明显改善。经济和社会事业加速发展，使人民群众得到更多实惠。城市居民人均可支配收入达到7383元，增长9.7％；农民人均纯收入达到3022元，增长12.3％；城乡居民储蓄存款余额达到611亿元，增长10.7％。出台了最低工资保障规定，初步建立了城镇企业工资正常增长机制。全社会商品零售总额完成192.1亿元，增长16.2％。城市居民用于住房、交通通信、教育文化等方面的人均支出分别增长22％、18％和 36.4％。在人民生活水平普遍提高的同时，困难群众生产生活问题引起了政府的高度关注。投入扶贫专项资金2.04亿元，解决了16.4万贫困和低收入人口的温饱问题。建立了对困难群众和弱势群体“接对子”、“一对一”等多种形式的帮扶责任制，农村特困户、困难群众生活得到改善。全面落实各项救灾措施，筹措发放救灾款2053万元，救助灾民19.8万人次，确保了灾区群众的基本生活。

（四）社会事业全面发展，精神文明建设取得新成果

科技体制改革稳步推进，科技创新能力得到提高。重点加强了5个企业技术中心建设，组织实施了34个科技攻关项目和高新技术产业化项目，科技进步对工农业发展的贡献率分别达到45％和 51％。农村中小学危房改造和布局调整成效显著，“双基”达标进一步巩固提高，农村普通中学规范化建设步伐加快，高教、职教、成教等工作取得新进展，沧州医专新校区一期工程竣工并投入使用。文学艺术、社会科学、新闻出版、广播电视等事业不断发展，优秀文化得到弘扬，《童声与二胡·七彩童年》、《 西河大鼓·俺村的新事实在多 》等一批文化创作精品在全国获奖。竞技体育取得新成绩，在首届国际传统武术比赛中，我市选手参加了三个项目的比赛，并获得了全部六枚金牌。成功举办了第五届全民健身运动会，人口与计划生育责任目标全面完成。加强了疾病防控、农村卫生和环境卫生建设，市县两级共建设疾控中心14个，进一步健全了疾病预防控制网络。思想道德建设不断加强，群众性精神文明创建活动深入开展。“双拥”共建、国防教育和国防后备力量建设取得新进展，征兵工作和优抚安置政策得到较好落实，驻沧部队在我市发展和建设中发挥了积极作用。

（五）民主法制建设和政府自身建设进一步加强

坚持定期向市人大及其常委会报告工作、向市政协通报情况，认真听取人大代表、政协委员、各民主党派和工商联提出的意见和建议，依法办复有关建议和提案。政府自身建设得到加强，依法行政水平有了新的提高。坚持依法决策，推行民主决策。进一步完善了重大问题集体决策制度、专家咨询制度、社会公示制度和社会听证制度，特别是对水价、气价、供暖等关系民生的调价项目，实行社会听证和专家咨询论证，维护了群众利益。深入贯彻《行政许可法》，开展行政效能监察，持之以恒地抓好环境建设。全面实施了承诺践诺、首办负责、投诉受理、责任追究等工作制度，政府及各部门服务意识、服务水平有了新的提高，从政行为进一步规范。深入开展廉政建设和反腐败斗争，审计违规资金7.8亿元，查处大案要案454件，挽回经济损失860万元。完善村民自治，推行社区自治，社区建设、基层民主政治建设扎实推进。高度重视人民群众来信来访，加大人民内部矛盾纠纷的排查调处力度，逐步完善维护稳定的长效机制，正确处理和化解人民内部矛盾。强化社会治安综合治理，深入开展“严打”整治斗争，保持了治安形势的总体平稳和社会秩序的进一步好转，为全市经济社会发展创造了稳定和谐的社会环境。

回顾过去一年来的工作，有六点深刻体会：一是必须始终保持高度的政治责任感，咬定发展不放松。只有时刻铭记并牢牢把握发展第一要务，才能专心致志地思富民之策、兴为民之举、办利民之事，真正代表沧州人民的根本利益。二是必须始终保持积极的、有作为的姿态。只有保持一股胜不骄、败不馁，勇于开拓、奋发有为的精神和气概，才能鼓舞士气，振奋精神，创造条件，求得发展。三是必须始终保持清醒头脑，准确把握机遇。只有时刻保持清醒的头脑，善于分析形势，捕捉信息，把握动向，才能在思路上想深想透，在行动上坚定不移；只有善于在复杂多变的形势下，洞察并把握即将出现和已经出现的机遇，才能赢得发展的主动权。四是必须始终从沧州实际出发，理清思路，抓住重点，突破难点。只有理清思路，善于抓大事，攻难点，才能抓住主要矛盾，牵住牛鼻子，取得以点带面、活跃全局的工作效果。五是必须始终坚持科学发展观。只有坚持科学发展观，才能切实转变经济增长方式，实现速度与结构、质量、效益相统一；才能立足实际，发挥优势，营造特色，实现区域经济协调发展；才能统筹兼顾，实现经济与社会、城市与农村、人口与自然的协调发展。六是必须始终坚持党的领导。只有坚持党的领导，才能赢得广大人民群众的拥护与支持，才能充分调动方方面面的积极性。我们坚信，在中共沧州市委的正确领导下，只要我们牢牢把握发展这个第一要务，就一定能够凝聚全市人民的智慧和力量，把沧州建设的更加美好。

各位代表，过去一年，我市经济和社会发展取得了令人鼓舞的好成绩，是沧州发展史上浓墨重彩的一笔。成绩来之不易，这是市委正确领导、市人大依法监督、市政协积极参政议政的结果，是我们应对挑战、锐意进取、实施以快赶强战略的结果，更是全市人民团结奋斗、顽强拼搏的结果。在此，我代表市人民政府，向辛勤工作在各条战线上的全市各族人民，向给予我们支持与监督的人大代表、政协委员、各民主党派、工商联、无党派人士，向大力支持我们工作的驻沧单位、人民解放军和武警部队，以及所有关心、支持沧州发展的各界人士，表示崇高的敬意和衷心的感谢！

各位代表，经济和社会发展的好形势，增强了我们继续前进的信心和勇气。但我们也清醒地认识到，与全市人民加快发展的迫切要求相比，还有一些亟待克服的困难和问题，主要是：经济总量偏小，经济结构调整作为一项长期任务仍然需要倾注更多的精力；城市建设和管理还不能满足人民的生活需要；从根本上解决“三农”问题还有待于深入探索；对外开放和环境建设仍需进一步加强；国企改革任务艰巨，就业压力加大；水、电、煤、油、运等生产要素对经济社会发展的“瓶颈”制约还没有从根本上得到缓解；政府工作作风有待进一步改进。以上这些，是发展进程中绕不开的矛盾，必须用发展的办法去克服和解决。

二、 2005年政府工作的指导思想、任务目标

2005年是承上启下，为“十一五”打好基础的关键一年。政府工作的指导思想是：以邓小平理论和“三个代表”重要思想为指导，全面贯彻党的十六大和十六届三中、四中全会精神，高扬“树正气、讲团结、求发展”和“创业、干事、为民”的主旋律，加强执政能力建设，以科学发展观统揽工作全局，抢抓机遇，加快发展，紧紧抓住对外开放、项目建设、民营经济、国企改革四项重点工作，进一步优化产业结构，扩大经济总量，提高经济效益，推进新型工业化、农业产业化、城市现代化进程。加强政治文明、精神文明建设，加速发展社会各项事业，实现全市经济社会全面、协调、可持续发展。基于这一指导思想，2005年的任务目标是：地区生产总值增长12％以上，全社会固定资产投资增长18％以上，全部财政收入增长10％以上；城市居民人均可支配收入增长8％，农民人均纯收入增长6％。

要实现以上任务目标，我们必须在工作基调、战略性举措等涉及我市经济社会发展的几个重大问题上形成共识。

把“更快、更好”作为工作基调。加快发展是解决前进道路上一切矛盾和问题的关键。深化改革过程中利益关系的调整、增加城乡居民收入、改善人民生产生活条件、促进经济与社会的协调发展等等，都有赖于加快发展。落实科学发展观，核心是发展，因此，我们要把实现沧州经济社会更快、更好的发展作为工作基调。一方面，要保持一个较高的增长速度。国家加强宏观调控不是搞一刀切，是有保有压、有抑有扬。只要我们的项目符合国家产业政策，消耗低、污染小、效益高、前景好，能搞多大就搞多大，能发展多快就发展多快。另一方面，更加注重增长的质量和效益，使速度与结构、质量、效益相统一。更加注重统筹协调发展，把发展建立在提高效益、优化结构、保护生态环境的基础之上，保持发展的全面、协调和可持续。当前，我们正面临一个新的发展机遇：全球性经济复苏和产业结构调整步伐进一步加快；我国经济处于新一轮增长的上升期；珠三角、长三角投资向内地转移；国家已正式将沧州列入京津冀都市圈，省委、省政府对沧州的发展寄予厚望，全市干部群众渴望大发展、快发展，等等。而且，我们也具备了加快发展的有利条件：密集的陆路交通网，黄骅大港的建成通航，使我们的区位优势和交通优势更加突出；历届党委、政府和全市人民的不懈努力，使我们的产业基础日益雄厚，经济实力日渐增强，发展的原动力更加强劲。只要我们把握住“更快、更好”的发展这个基调，以积极的、有作为的姿态，抓住机遇，扬长避短，发挥优势，就一定能够在加快发展的征途上创造新业绩，跃上新台阶，实现新跨越。

用“五个一批”统揽工业强市。发达的工业和雄厚的产业是城市现代化的基石。必须坚定不移地把优先发展工业作为强市之本、富民之基，以“五个一批”为统揽，加快建设工业强市。第一，以“五个一批”为目标，加速工业经济总量扩张。进一步完善“五个一批”规划，充实细化“五个一批”内容，将效益好、有特色、有前途的产业基地、骨干行业、重点企业及村镇纳入“五个一批”。加大对具体项目的扶持力度，倾力打造 “五个一批”，壮大工业经济规模。第二，以亿元以上工业项目建设为载体，构筑雄厚的产业支撑。加快谋划建设一批消耗低、污染小、效益高、符合国家产业政策的亿元级、十亿元级工业大项目，并以此带动相关产业发展。第三，以国企改革为切入点，增强核心竞争力。通过出售、出让、兼并、租赁、合资、合作等多种方式，盘活存量资产，加快企业改组改造步伐，激发企业内在活力，使一批企业走出生产经营困境。加强与国内外大集团、大企业合作，鼓励民资、外资参与国企改组改造，加速实现企业产权多元化。进一步完善企业经营机制，加强内部管理，提高经济效益。加快企业信息化建设，推动企业技术、管理升级，提升市场竞争力。

以“重中之重”看待三农问题。有效解决“三农”问题直接关系到全面建设小康社会宏伟目标的实现，必须以“重中之重”看待三农问题，围绕农业增效、农民增收，全面发展农村经济。第一，深度推进“四大产业，一大工程”，加速农业产业化进程。在发展粮食、棉花等传统农业生产的基础上，继续抓好草、枣、畜、菜“四大产业”和旱盐碱地开发“一大工程”。大力培植壮大农业产业化龙头企业，拓展沛然世纪、东方果品、河北乡谣、小洋人等龙头企业发展空间，推广市场连企业，企业连基地，基地连农户的生产、流通、销售模式，带动更多农户进入产业化发展链条。加强农业专业合作组织建设，强化面向农户的技术支持、市场预测、信息共享等各项服务，提高农业综合效益。第二，积极推行农业标准化生产。突出良种化、优质化、标准化和品牌化四个重点，大力引进农业新品种，加快优质小麦、优质无公害小杂粮、优质棉花和优良林业苗种基地建设。积极推进品牌认证、整合工作，鼓励和引导名优新特农产品申报省级、国家级名牌农产品，培育和壮大沧州特色农业品牌。第三，抓好农业项目建设，促进农村全面协调发展。围绕梨、枣、肉、菜、草以及水产品、优质粮等优势资源，谋划建设一批技术含量高、市场前景好的农副产品深加工项目；围绕优势农副产品外销，建设发展一批大型专业批发市场；围绕解决水资源短缺问题，实施一批节水灌溉、海水淡化、河道堤防整治等节水、改水和水利工程项目；围绕增加农民现金收入，大力发展劳务输出组织和劳务中介机构，促进劳务输出和农村富余劳动力向非农领域转移。

将“沿海临港”视为最大优势。黄骅大港的建成通航，使沧州成为名副其实的沿海开放城市。沿海临港是沧州的优势所在、希望所在和潜力所在，必须立足发挥沿海临港的独特优势，做好港口经济这篇大文章。第一，促进生产要素向沿海聚集。突出港口带动作用，吸引要素资源向沿海区域流动，加快形成以港口为龙头，以临港地带为依托的物流、人流、资金流聚集地，做大做强临港经济和海洋经济。第二，加快临港工业基地和港口海运基地建设。重点发展盐化工、石油化工、煤化工、精细化工、船舶修造等工业项目。积极发展临港出口加工业，引进开发“两头在外”的加工贸易项目， 重点发展粮油食品、皮革制品、原料化工及钢铁、木材、煤炭加工等大进大出型临港加工业项目。下大力气抓好港口建设，完善地方杂货码头、渔港码头和液体化学品码头功能，加快港口物流区建设，大力发展航运业和现代物流业。第三，积极发展海洋渔业和海洋新兴产业。以水产品养殖、捕捞、加工为重点，加快发展水产养殖业 ，引进一批海产品保鲜、保活、精深加工和综合利用项目，着力打造环渤海地区重要的水产品精深加工基地。加快发展海水淡化、风力发电、海洋生物医药、海洋保健食品等海洋新兴产业。

三、攻坚克难，强力突破四项重点工作

紧紧抓住对外开放、项目建设、民营经济、国企改革四项重点工作，以重点工作的突破活跃经济发展全局。

（一）进一步扩大对外开放。抢抓新一轮全球生产要素优化重组和产业转移的重大机遇，加速构建开放型经济新格局。 第一，创新招商方式，提高利用外资水平。认真总结、改进和创新招商方式，坚持专业化、小团组、多批次、一对一的原则，组织开展、积极参与各类招商活动，增强招商工作的针对性和实效性，力争在引进世界500强和国际知名企业方面取得突破。第二，开展好“日韩招商年”活动。精心组织好专项经贸洽谈活动，搞好项目包装推介，在化工、线缆、汽车配件、电子通讯、纺织服装、农产品加工等领域谈成一批合作项目。第三，加强招商载体建设。以园区招商、产权招商、产业链招商、以商招商为重点，着力引进一批产业关联度高、辐射带动作用强的大项目、高新技术项目。倾力抓好己内酰胺、津汕高速、城市燃气、华峰热电等利用外资500万美元以上的项目。第四，努力扩大外贸出口。调整优化出口结构，提高传统出口产品竞争力，进一步扩大裘皮制革、针织毛衫、机电、特色农产品的出口创汇能力。第五，搞好跨区域经济合作。深化对“环渤海、环京津”内涵和外延的把握，在打好京津牌的同时，主动承接先进地区，特别是珠三角、长三角地区产业转移项目，引进一批符合国家产业政策、成长性良好的劳动密集型产业。

（二） 加强项目建设。坚持以大项目带动大投入，以大投入促进大发展，力促投资总量、项目质量跃上新台阶。第一，全面落实项目谋划、建设责任制。健全投资决策和项目法人约束机制，对重大项目组建项目公司，建立项目法人制，实行企业化运作。着力抓好项目库建设，重点谋划、筛选、储备一批规模大、技术含量高、产业关联度强、聚集效应明显的立市兴县大项目。第二， 突出抓好大项目建设。抓好40万吨PVC、液体化学品码头及罐区、 5万吨TDI、12万吨己内酰胺、沧州炼油厂和中石油华北石化分公司扩能改造、肃宁帝贤纺织、泛美服装等工业生产项目，打造强有力的工业支撑体系；抓好国华沧东电厂、华峰热电、海挡延伸造地、沧黄、津汕、阿深和沧保高速公路等能源、交通项目，夯实发展基础；抓好燃气公司改扩建、大浪淀二期、市区水系景观、华北商厦扩建、新华、运河两区综合改造等城市建设和服务业建设项目，进一步完善城市功能；抓好环核苷酸天然提取等高新技术项目，加快农业产业化进程；抓好市疾控中心、沧州学院、全民健身中心等社会事业项目建设，促进经济社会协调发展。

（三）壮大民营经济。第一，强化政策扶持，优化发展环境。着重抓好扶持民营经济发展相关政策措施的落实，在投资融资、税收、土地使用和对外贸易等方面，让民营企业与其他企业享受同等待遇，激发民营企业家的创业热情。健全和完善民营企业信用担保、诚信评估、人才培训、法律服务体系，切实解决民营企业融资困难、信息不灵、人才缺乏等问题。第二，鼓励民营企业提升整体素质。坚持大中小并举，在扶持一批主业突出、管理先进、核心竞争力强的优势企业的同时，按照产业布局，引导中小民营企业入园进区经营，加快形成一批区域性特色产业集群。充分发挥现有优势产业和优势企业在对外合作中的作用，全面加强与国内外大集团、大企业嫁接合作，实现强强联合，壮大产业基础。大力推进民营企业制度创新、技术创新和管理创新，促进民营企业向科技型、外向型、集团化方向发展。

（四）强力推进国企改革。按照今年上半年市县属国有资本基本退出的总目标，加速推进国企改革，实现国有资产保值增值和国有经济布局的调整。 第一，加大市县属国有企业改革力度。 以建立现代产权制度为核心 ，以劳动关系调整为关键，因企制宜、一企一策，强力推进国有企业改革。重点抓好沧化集团、地铁集团等大中型企业的产权改制，加快发展国有资本、集体资本和非国有资本参股的混合所有制经济。第二，稳步推进企业关闭破产。在妥善处理化机集团破产后续工作的基础上，加快沧州啤酒厂、市造纸厂等企业的破产申报和程序操作。第三，完善国有资产监督管理体制，健全公司法人治理结构，加强对国有重点企业经营业绩考核。完善国有资产运营公司职能，引导和促进国有资本向优势行业和优势企业有序集中。

四、更加注重发展的薄弱环节

抓好发展的薄弱环节，是落实科学发展观，实现统筹发展和“更快、更好”发展的重要保证。

（一）加强城市建设，加快城市化进程。第一，加强中心城市建设和管理，提升中心城市辐射带动功能。 以建设现代化滨海港口城市为目标，坚持繁荣沧州市区与建设港口新城协调同步，打造沧州中心城市“哑铃型”结构特色。坚持“文明城市”、“卫生城市”、“园林城市”三城同创，完善市区总体规划、各专项规划和控制性详规，加大市容环境整治力度。加强城市建设，努力营造总体风格协调一致，单体建筑个性突出的建筑特色，重点实施一批强化载体功能、提升城市品位、地方特色浓郁的标志性、公益性和旧城改造项目，推进市区—开发区—港城区组团式发展。 第二，创新城市管理体制，搞活城市经营。深化投融资体制改革和市政公用事业改革，全面开放市政公用资本市场、经营市场和作业市场，鼓励和吸引社会资本、境外资本投资城市基础设施和市政公用事业。加强城市资源综合开发，搞好土地储备和城市无形资产经营，有效盘活存量资产。完善城市管理配套政策和相关法规，相对集中行政处罚权，解决城市建设征地难、拆迁难、建设阻工等问题。第三，进一步优化城镇发展布局，推进城市化进程 。加速构建以沧州市为中心，以任丘、河间、黄骅、泊头为次级中心城市，以县城为重要节点的城市发展格局。加快制定完善土地、户籍、劳动就业、文化教育、医疗卫生、计划生育等配套政策，为加速城市化提供政策支持。

（二）坚持统筹发展，壮大社会事业。在发展经济的同时，更加注重社会事业发展。第一，充分发挥“科教”在“兴市”中的作用，搭建科技平台，增强科技创新能力，提高科技对经济增长的贡献率。着力抓好生产力促进中心、星火科技专家大院、化工中试基地和农业科研中试基地建设。整合教育资源，提高教育质量，大力培养实用型人才。创新人才工作机制，抓住培养、引进、使用三个环节，大力营造优秀人才脱颖而出、充分施展才能的良好环境。第二，实施武术、杂技等精品文化开发工程，打造沧州特色文化品牌，培育发展一批文化企业集团。完善公益性文化设施，活跃群众业余文化生活。第三，建立健全疾病防控体系、疫情信息网络体系和医疗救治体系，提高公共卫生服务水平和应对处置突发性公共卫生事件的能力。加大对农村卫生事业的投入，加强农村三级医疗预防保健网络建设，搞好青县、黄骅两县市新型农村合作医疗制度试点。第四，搞好环境保护，加强人口与计划生育工作，稳定低生育水平，提高出生人口素质。深入开展国防教育、国防动员、民兵、预备役和双拥共建活动。依法管理宗教事务，大力发展民族经济，促进民族团结。高度重视史志档案、妇女儿童、残疾人和老龄工作，大力发展体育、新闻出版、广播电视等各项社会事业。

（三）谋划大事抓大项目，增强市本级实力。做好市本级工作，不仅具有辐射带动作用，而且具有示范引导作用。2005年，根据方方面面的意见，市本级集中精力抓四件大事。第一，加快“四区一园”建设，搭建市本级发展平台。即加快推进沧州开发区、港城开发区、临港化工园区、沧州新区和神华万亩工业园建设，使之成为市本级快速发展的平台和重要的经济增长极。沧州开发区：以项目建设为主，城市建设为辅，发展成为沧州东部新兴工业区和生活区。目前要加强软硬环境建设，以低商务成本和高投资回报吸引投资者创业发展，加快机械、轻工、医药、信息产业发展，使之逐步形成产业集群。港城开发区：落实中心城市发展总体规划，坚持项目建设与城市建设并重，加快构建市区——港城“哑铃型”中心城市格局。要以项目建设拉动城市建设，以城市建设促进项目建设，立足港口优势，积极发展以煤、电为基础能源的加工制造业、物流业两大支柱产业，带动仓储、航运、旅游、房地产等相关产业发展。临港化工园区：充分发挥液体化学品码头和土地资源优势，以建设北方大型化工产销基地为目标，围绕盐化工、石油化工、煤化工、精细化工发展做大文章，深度开发上、下游产品，拉长产业链条，成为市本级重要的工业区和财力的支撑点。上述三区在工作中都要坚持以开放为动力，以项目为抓手，2005年要在利用外资超千万元的大项目上有新的突破。深入谋划、有序开发沧州新区。积极配合神华集团，加快建设神华万亩工业园。第二，集中力量实施好以新旧城区结合部为主攻方向的城市建设工程。按照“全面推进，集中突破，营造特色”的总体思路，加强西外环东侧，以及由西外环向东延伸的解放西路、迎宾西路、南环西路等区域建设项目的谋划、设计、开发和建设，集中精力打造现代城市新景观，使之成为全市城市建设的精品区。第三，倾力抓好三组立市大项目，构筑沧州未来产业发展格局。建设 40万吨PVC、5万吨TDI、12万吨己内酰胺三组大化工项目意义重大。这些项目有的已经开工，有的即将开工，有的正在抓紧前期工作，加上配套设施，投资近百亿元，如果得以顺利实施，对于构筑沧州未来发展战略支撑，提升工业发展整体水平，以及扩充市本级实力都具有十分重要的意义，必须倾全市之力，抓好这三组大化工项目。第四，以项目谋划为主线，编制好“十一五”规划 。“十一五”是沧州经济社会发展的重要时期，也是全面建设小康社会的关键时期，谋划、编制好“十一五”规划意义重大。“十一五”规划编制要体现“积极稳妥、求实创新、突出特色、便于操作”的原则，体现三个关注：一是关注人民群众的发展需求，让人民群众从发展中得到更多实惠。二是关注经济与社会的统筹协调发展。三是关注人与自然的和谐共生。要从生产力布局、资源开发、环境保护、发展循环经济等方面统筹考虑，促进经济与社会发展协调同步，人与自然和谐共生，走可持续发展道路。最根本的，还是要谋划一批足以支撑起“十一五”发展的骨干项目。

（四）抓好生产要素运行调节。有效解决经济社会发展中的瓶颈问题，是保证健康持续发展的重要措施。第一，实行节水、调水和开发利用相结合，进一步强化对水资源的统一管理，积极发展循环经济，鼓励实行水循环处理和重复利用。在全社会大力倡导节水意识，推广使用节水、集水建筑设施。有计划关闭市区及临港化工园区自备井，限制超采地下水。 加快建设县市和工业园区污水处理厂，推进水资源的深度开发利用。做好引水河渠及境内水库、洼淀等蓄水设施的维修与管护。加快南水北调“两渠十库”工程的开工建设，启动运河绕城规划，确保水资源引得来，留得住，用得好。第二，积极应对煤、电、油、运供应紧张状况，加强与有关方面的联系与沟通，强化协调调度，力求最大程度地保证供应，确保企业生产经营秩序和群众生活不受影响。

五、加强执政能力建设，打造新时期有为政府

加强执政能力建设，必须主动适应时代发展要求，努力提高政府领导、组织和管理经济社会发展的能力，让人民群众从发展中得到更多的实惠。

（一）优化经济发展环境，维护公平正义 。 一是优化政务、政策环境。着眼服务经济发展， 加强行政效能建设，努力提高办事效率和工作质量。全面落实《关于进一步优化发展环境的若干规定》，严肃查处“中梗阻”行为，确保各项优惠政策落实到位。适应形势发展，创新政策优势，不断完善支持企业改革改制、扩大开放、重点企业保护和民营企业扶持等相关政策。二是优化市场环境。严厉打击一切干扰企业生产经营秩序、重点项目建设，以及侵害企业法人、外地客商合法权益的行为；严厉打击欺行霸市、强买强卖、垄断市场等不法经营行为；严厉打击假冒伪劣、坑蒙拐骗、合同欺诈等经济领域的违法犯罪行为。 三是优化法制环境。深入贯彻《行政许可法》，完善行政执法工作标准，严格执法权限和执法程序，规范行政执法行为。实行重大处罚事项报告制度，落实执法责任制和行政过错追究制，用硬制度进行强约束，坚决制止不作为和乱作为现象。

（二）落实社会保障，构建和谐沧州。 做好社会保障、就业再就业、社会救助和扶贫开发工作，是提高人民群众生活水平的重要措施，是实践“三个代表”重要思想的具体体现，也是正确处理改革发展稳定关系的重要结合点。一是切实落实各项社会保障措施， 努力做好社会保障工作。确保国有企业下岗职工基本生活保障金、失业人员失业救济金和离退休人员养老金足额发放到位。完善城市低保制度，逐步建立农村低保制度，切实增强社会保障功能 。二是努力做好特困群众救助和扶贫开发工作。加快建立扶助困难群众的长效机制，完善特困户救助制度。大力实施“送温暖工程”，广泛开展资助贫困家庭学生就学活动。加大扶贫开发力度，着力解决农村贫困人口的温饱问题。三是千方百计促进就业和再就业。加强下岗、失业职工培训，提高劳动技能。认真落实再就业优惠政策，加快劳动力市场建设，规范职业中介组织从业行为，完善就业服务体系。四是进一步维护社会稳定。加强社会治安综合治理，建立健全社会预警体系，提高保障公共安全和处置突发事件的能力，依法打击各种犯罪活动，保障人民生命财产安全。认真抓好安全生产，积极做好人民内部矛盾排查调处，维护社会正常生产生活秩序。

（三）拓宽民主渠道，集中群众智慧。充分尊重和保障人民民主权利，拓宽民主渠道，扩大民主监督，是社会主义政治文明建设的重要内容。一是改革和完善决策机制，推进政府决策的科学化、民主化。对涉及全市经济社会发展全局的重大事项，广泛征求意见，集中群众智慧，实行科学决策、民主决策；对专业性、技术性较强的重大事项，认真组织专家论证，进行技术咨询和决策评估；对与群众利益密切相关的重大事项，实行公示、听证制度，维护群众利益。二是各级政府要自觉接受同级人大及其常委会的监督，坚持向人大及其常委会报告工作制度，支持人民政协履行职责，加强与各民主党派、工商联、无党派人士的联系，充分尊重和发挥他们在法制监督、政治协商和参政议政中的作用。三是加强基层民主政治建设，扩大基层民主。进一步完善基层政权、基层群众性自治组织、企事业单位的民主管理制度。推进政务公开、村务公开，特别是农村财务、征用土地等直接涉及农民利益事务的公开。健全职工代表大会和其他形式的企事业单位民主管理制度，保证人民群众依法行使选举权、知情权、参与权和监督权。

（四）扑下身子，埋头苦干，为沧州发展做实事。大力弘扬求真务实之风，把抓落实、求实效、办实事体现到政府工作的各个方面。一是建立主体清晰、责任明确、权责统一的执行责任体系，完善考核监督机制，保证各项决策快速准确落实。加强政府机关、职能部门和公务员队伍建设，加大宣传、教育力度，通过领导机关、领导干部率先垂范，形成自上而下抓落实的工作作风和良好工作习惯。二是把推动沧州更快更好的发展作为最大的政绩工程。提高执政能力，关键是提高领导发展的能力。要把政绩与增强区域经济实力，提高人民生活水平紧密联系起来，不遗余力地去奋斗，去创业，让人民群众享受发展的成果。三是把出实招、办实事作为加强执政能力建设的最佳体现。加强执政能力建设不是空洞的口号，最终要体现到实际行动上。坚持出实招、办实事，谋划一批能够振奋人心的大项目，建设一批有实力、有影响，能够惠及沧州几代人的大工程，把加强执政能力建设体现到具体工作中，落实到实际行动上。

各位代表，沧州正面临着十分难得的发展机遇，让我们紧密团结在以胡锦涛同志为总书记的党中央周围，认真落实党的十六大、十六届三中、四中全会和省委六届六次全会精神，围绕再造一个新沧州的战略目标，在中共沧州市委的坚强领导下，以高昂的斗志锐意改革，以饱满的热情致力创新，以求实的精神促进发展，努力把改革发展中的难题一道一道地破解，把造福人民的实事一件一件地办好，让全市人民在沧州的改革发展中真正得到实惠！
